# Supplementary material for: Collaborative intelligence in AI: Evaluating the performance of a council of AIs on the USMLE
Source: PLOS Digit Health. 2025 Oct 9;4(10):e0000787. doi: 10.1371/journal.pdig.0000787 (PMC12510544; doi:10.1371/journal.pdig.0000787)
Supplement: S2 File — (ZIP) [file pdig.0000787.s002.zip › S2 Data Raw data files containing unprocessed question inputs and LLM outputs including transcripts of deliberations/STEP3_Question-115-1_Type-MC-NJ.docx]

**Facilitator** **[Round 1]:** A 47-year-old man comes to the emergency department because of a 3-day history of increasing pain, tenderness, and swelling near the fingernail of his right index finger. He rates the pain as an 8 on a 10-point scale. He has not had fever or chills, and he has not sustained any trauma to the finger. Medical history is unremarkable and the patient takes no medications. He appears generally well and is not in distress. Vital signs are temperature 37.1°C (98.8°F), pulse 72/min, respirations 14/min, and blood pressure 120/80 mm Hg. Physical examination of the right hand discloses swelling, erythema, warmth, and tenderness of the periungual region of the right index finger. There is a mildly fluctuant area near the nail edge. The remainder of the physical examination discloses no abnormalities. Which of the following is the most appropriate next step in management? (A) Incision and drainage (B) Oral cefazolin therapy (C) Topical neomycin therapy (D) Warm-water soaks

**AI_C:** 1) RESTATING THE QUESTION: The question asks which of the presented options would be most appropriate to treat a 47-year-old man's symptoms, which involve increased pain, swelling, and tenderness around his right index finger's fingernail. This patient hasn't had any fever or chills and hasn't experienced any trauma to the affected finger. His medical history is unremarkable, and he doesn't take any medications. The physical examination revealed swelling, erythema, tenderness and warmth of the periungual region of the right index finger, with a mildly fluctuant area near the nail edge. 2) PLANNING: Generally, these symptoms may suggest a condition called paronychia, which is an infection of the tissue surrounding the nail. Options (A) Incision and drainage, (B) Oral cefazolin therapy, (C) Topical neomycin therapy, and (D) Warm-water soaks are suggested as potential treatments. We need to question the efficiency and relevancy of each of these interventions, considering the severity, presence of fluctuation, and infecting organism in this case. 3) CLINICAL REASONING: Incision and drainage is a typical first-line treatment for abscesses or localized infections with purulent discharge or fluctuation. Oral cefazolin therapy might be considered if a systemic infection is suspected, but as of the information given, the patient doesn't exhibit systemic symptoms like fever or chills. Topical neomycin therapy might be applied to minor, superficial skin infections, but it's not usually effective for deeper tissue infections. Warm-water soaks can help alleviate symptoms but do not directly address the underlying infection. 4) CONCLUDING: Upon analyzing these options, considering that the patient doesn't have systemic symptoms and the infection seems to be localized around the nail (periungual infection), incision and drainage seems to be the most appropriate first-line approach as it targets the core issue, deep tissue infection, specifically when a fluctuant area is present. 5) RESPONSE: The most appropriate next step in the management of this patient would therefore be (A) Incision and drainage.

**AI_D:** RESTATING THE QUESTION: The patient, a 47-year-old male, has presented with symptoms primarily localized to the right index finger, specifically the periungual region - the area surrounding a nail. Key details indicate swelling, erythema (redness), warmth, and tenderness, along with a slightly fluctuant area near the edge of the nail. Despite the absences of fever and trauma, the patient experiences significant pain, rated 8 out of 10. He appears generally well and presents no other abnormalities based on a physical examination. Given these circumstances, we are to identify the best course of action from four treatment options. PLANNING: To ascertain the most suitable course of action, we need to diagnose the condition first. Based on the presented symptoms -pain, swelling, redness, warmth, and fluctuant area near the nail edge without systemic symptoms such as fever-, a diagnosis of paronychia, a common soft tissue infection surrounding the nail, can be safely assumed. These symptoms are consistent with an abscess formation, which is a common complication of paronychia and usually causes a tender, fluctuant mass to form. From here, the treatment options given can be reviewed and evaluated in the context of the possible diagnosis. CLINICAL REASONING: For a paronychia, particularly when an abscess is involved, initial treatment typically involves drainage. This is because antibiotics alone often cannot penetrate abscesses effectively, and without drainage, the infection might not resolve. So, among the offered choices (A) incision and drainage, seems most feasible and directly addresses the most possible assumed diagnosis. Oral or topical antibiotics (choices B and C), are usually reserved for severe or persistent cases of bacterial paronychia that have not responded to initial treatment. But as there are no signs of infection spread, systemic antibiotic therapy is not the first line of action. Warm-water soaks (choice D) - though often used as a supportive measure, particularly in the early stages of the condition, when abscess formation is absent - would provide relief but would not treat the abscess directly. CONCLUDING: Considering the given symptoms and their correlation with a symptomatic paronychia with abscess formation, the most appropriate next step in management would involve addressing the abscess directly due to the impeded ability of antibiotics to penetrate and resolve it. RESPONSE: The most appropriate next step in managing this patient's condition should be (A) Incision and drainage.

**AI_E:** RESTATING THE QUESTION: A 47-year-old gentleman shows up in the emergency department with a 3-day history of escalating discomfort, tenderness, and inflammation near the nail of his right index finger. His pain scale is 8 out of 10, though he does not exhibit symptoms of fever or chills and did not experience any trauma to the said finger. No pertinent medical history has been noted, and the patient doesn't take any medications. His overall appearance is seemingly healthy and there is no evidence of distress. Upon physical examination, there seem to be inflammation, redness, increased warmth, and tenderness around the nail area of the right index finger. There is a slightly fluctuating spot near the edge of the nail. The remainder of the physical examination did not uncover any other abnormal findings. The most appropriate next step in management is being determined. PLANNING: Possible assumptions would be that the patient might have either started developing an infectious process around his fingernail (described as periungual), specifically a paronychia, or an inflammation of the hand unrelated to infection. Although trauma has not been reported, it could also be an indication of an abscess formation. Based on these scenarios, we would have to identify the clinically prioritized assumption. It seems logical to assume that an infectious process might be underway considering the reported symptoms. Therefore, the focus should be on managing this suspected infection. CLINICAL REASONING: Paronychia is an infection of the skin around the fingernails, often caused by bacterial (commonly Staphylococcus aureus and Streptococcus pyogenes) or fungal infections. It typically presents with redness, swelling, and pain in the area around the nail, similar to the symptoms exhibited by this patient. A fluctuant area is indicative of a collection of pus. Non-fluctuant abscesses are typically treated with warm soaks and oral antibiotics. However, paronychias that are fluctuant are indicative of an abscess and the pus needs to be released in order for the body to heal. CONCLUDING: Given the clinical picture suggestive of a fluctuant paronychia, the first line of treatment involves drainage of the abscess. This is achieved by making an incision and draining the collected pus, thus reducing the pressure that causes the intense pain. Oral antibiotics, if required, are only adjunctive to surgical drainage. Thus, the necessary step for effective treatment in this case would be incision and drainage. RESPONSE: Thus, the most appropriate next step in management for this patient with a fluctuant paronychia is (A) Incision and drainage. This treatment will help to effectively eliminate the subungual abscess, subsequently reducing the pressure-induced pain, and generally encouraging better healing.

**AI_B:** 1) RESTATING THE QUESTION: This query seeks an optimal method to manage a 47-year-old man's condition; he's been experiencing ongoing pain, redness, warmth, and swelling in the region near the fingernail of his right index finger for the past three days, with an area of slight fluctuation by the nail edge. Despite these symptoms, the patient does not report any fever or chills and denies any history of trauma. All vital signs and other aspects of his physical examination are within normal range. The periungual region is specifically near or around the nail of a finger or toe. 2) PLANNING: There are a few potential diagnoses to consider for this case based on his symptoms, including cellulitis and paronychia (an infection of the skin surrounding a fingernail or toenail), which are the main considerations here. Both conditions present with redness, swelling and pain; however, the presence of a fluctuant area, generally indicating abscess formation, is more suggestive of paronychia. Our primary concern is whether the infection necessitates surgical intervention (in case of an abscess requiring drainage), or could be managed conservatively using antibiotics or warm-water soaks. 3) CLINICAL REASONING: Paronychia typically results from disruption in the protective barrier between the nail fold and the nail plate, allowing introduction of bacteria, leading to infection. Despite the absence of a reported trauma, simple actions like aggressive nail-biting or manicures might cause such disruption. The presentation is consistent with paronychia, especially considering the fluctuant area near the nail edge, suggesting an abscess, which is essentially a walled-off collection of pus. Antibiotics (either oral or topical) can treat the infection but will not necessarily address an abscess if one is present. To definitively manage an abscess, incision and drainage are necessary to remove the collected pus. Warm-water soaks, on the other hand, while useful for mild paronychia without abscess formation, are unlikely to be sufficient for this case. Hence, the next step should be to alleviate the patient's symptoms and eliminate the infection source by draining the abscess. 4) CONCLUDING: Utilizing step-by-step clinical thought processes, we can deduce that incision and drainage should be the next step in managing this patient's symptoms and condition. While antibiotics can treat the bacterial infection element, they wouldn't adequately address the likely abscess as suspected due to the fluctuant area near the nail edge. Warm-water soaks could potentially aid in reducing the inflammation, but again, wouldn't sufficiently deal with the suspected abscess. 5) RESPONSE: The most appropriate next step in management, based on the clinical picture, should be (A) Incision and drainage.

**AI_A:** RESTATING THE QUESTION: The patient, a 47-year-old male, has presented with a 3-day history of severe pain, tenderness, and swelling around the fingernail of his right index finger, but without a history of fever, chills, or trauma. The pain is rated as 8 out of 10. The area in the periphery of the nail is erythematous, warm, and tender, with a fluctuant area (a palpable fluid-filled cavity) nearby. His vital signs are normal, his medical history is unremarkable, and he takes no medications. The question is asking what would be the best next step to manage this patient's condition. PLANNING: Underlying assumption is that the patient's condition indicates a localized bacterial infection around the nail bed, known as paronychia, which is often due to Staphylococcus aureus, Streptococcus species, or anaerobic bacteria. An alternate assumption might be that the patient has a foreign body, a herpetic whitlow, a felon's tip abscess, or a developing septic arthritis. However, the presence of fluctuance, erythema, and tenderness involving the periungual area without preceding trauma suggest a bacterial paronychia. The lack of systemic symptoms like fever, chills, and inability to concentrate the symptoms to a single joint suggests against septic arthritis. The absence of a history of trauma, puncture, or penetrating injury reduces the likelihood of foreign body infection or a felon's tip abscess. Moreover, the absence of vesicles and the presence of fluctuance suggests against a herpetic whitlow. Given these considerations, the assumption that the patient's condition is due to a bacterial paronychia is valid to pursue. CLINICAL REASONING: Paronychia usually develops when a break in the skin allows bacteria to invade the paronychial fold, a skin fold touching the nail. Since the causative organism is likely to be S. aureus or Streptococcus species or anaerobic bacteria, appropriate treatment aims at eliminating the infection and providing symptomatic relief. The primary treatment of acute paronychia with ongoing abscess formation, as suggested by the presence of fluctuance, is incision and drainage of the abscess. Following drainage, warm soaks should be initiated to promote continued drainage. Antibiotic therapy is reserved for those who have signs of cellulitis or immunocompromised patients. CONCLUDING: Given the clinical presentation suggesting the patient has an acute bacterial paronychia with abscess formation, the best course of action would be to perform the incision and drainage of the abscess to release the built-up pus, followed by warm soaks to promote continued draining. Antibiotics are generally not needed unless there are signs of cellulitis or underlying immune-compromised status. RESPONSE: Thus, the most appropriate next step in management is to perform an incision and drainage (Choice A), which would relieve the built-up pressure and allow the infection to drain, leading to subsequent symptomatic relief.

**Facilitator** The consensus statement synthesizes the responses of AI_A, AI_B, AI_C, AI_D, and AI_E as follows: The patient, a 47-year-old male, presents with a 3-day history of severe pain, tenderness, and swelling around the fingernail of his right index finger. Despite the absence of fever, chills, or trauma, the pain is significant, rated as 8 out of 10. The physical examination reveals erythema, warmth, and tenderness in the periungual region of the right index finger, with a mildly fluctuant area near the nail edge. The patient's medical history is unremarkable, and he takes no medications. The symptoms suggest a localized bacterial infection around the nail bed, known as paronychia, which is often due to Staphylococcus aureus, Streptococcus species, or anaerobic bacteria. The presence of fluctuance, erythema, and tenderness involving the periungual area without preceding trauma supports this diagnosis. The lack of systemic symptoms like fever and chills, and the absence of a history of trauma, puncture, or penetrating injury, further validate this assumption. Paronychia usually develops when a break in the skin allows bacteria to invade the paronychial fold, a skin fold touching the nail. Given the likely causative organism, appropriate treatment aims at eliminating the infection and providing symptomatic relief. The primary treatment of acute paronychia with ongoing abscess formation, as suggested by the presence of fluctuance, is incision and drainage of the abscess. Following drainage, warm soaks should be initiated to promote continued drainage. Antibiotic therapy is reserved for those who have signs of cellulitis or immunocompromised patients. Therefore, given the clinical presentation suggesting the patient has an acute bacterial paronychia with abscess formation, the best course of action would be to perform the incision and drainage of the abscess to release the built-up pus, followed by warm soaks to promote continued draining. Antibiotics are generally not needed unless there are signs of cellulitis or underlying immune-compromised status. Thus, the most appropriate next step in management is to perform an incision and drainage, which would relieve the built-up pressure and allow the infection to drain, leading to subsequent symptomatic relief.
